# Subjective cognitive decline, mild cognitive impairment, and dementia - syndromic approach: recommendations of the Scientific Department of Cognitive Neurology and Aging of the Brazilian Academy of Neurology

**DOI:** 10.1590/1980-5764-DN-2022-S101PT

**Published:** 2022-11-28

**Authors:** Jerusa Smid, Adalberto Studart-Neto, Karolina Gouveia César-Freitas, Marcia Cristina Nascimento Dourado, Renata Kochhann, Breno José Alencar Pires Barbosa, Lucas Porcello Schilling, Márcio Luiz Figueiredo Balthazar, Norberto Anízio Ferreira Frota, Leonardo Cruz de Souza, Paulo Caramelli, Paulo Henrique Ferreira Bertolucci, Márcia Lorena Fagundes Chaves, Sonia Maria Dozzi Brucki, Ricardo Nitrini, Elisa de Paula França Resende, Francisco Assis Carvalho Vale

**Affiliations:** 1 Universidade de São Paulo, Faculdade de Medicina, Departamento de Neurologia, Grupo de Neurologia Cognitiva e do Comportamento, São Paulo SP, Brasil.; 2 Universidade Federal do Rio de Janeiro, Instituto de Psiquiatria, Rio de Janeiro RJ, Brasil.; 3 Hospital Moinhos de Vento, Porto Alegre RS, Brasil.; 4 Universidade Federal de Pernambuco, Centro de Ciências Médicas, Área Acadêmica de Neuropsiquiatria, Recife PE, Brasil.; 5 Instituto de Medicina Integral Prof. Fernando Figueira, Recife PE, Brasil.; 6 Pontifícia Universidade do Rio Grande do Sul, Escola de Medicina, Serviço de Neurologia, Porto Alegre RS, Brasil.; 7 Pontifícia Universidade do Rio Grande do Sul, Instituto do Cérebro do Rio Grande do Sul, Porto Alegre RS, Brasil.; 8 Pontifícia Universidade do Rio Grande do Sul, Programa de Pós-Graduação em Gerontologia Biomédica, Porto Alegre RS, Brasil.; 9 Universidade Estadual de Campinas, Faculdade de Ciências Médicas, Departamento de Neurologia, Campinas SP, Brasil.; 10 Hospital Geral de Fortaleza, Serviço de Neurologia, Fortaleza CE, Brasil.; 11 Universidade de Fortaleza, Fortaleza CE, Brasil.; 12 Universidade Federal de Minas Gerais, Departamento de Clínica Médica, Belo Horizonte MG, Brasil.; 13 Universidade Federal de São Paulo, Escola Paulista de Medicina, Departamento de Neurologia e Neurocirurgia, São Paulo SP, Brasil.; 14 Hospital de Clínicas de Porto Alegre, Serviço de Neurologia, Porto Alegre RS, Brasil.; 15 Universidade Federal do Rio Grande do Sul, Faculdade de Medicina, Departamento de Medicina Interna, Porto Alegre RS, Brasil.; 16 Universidade Federal de Minas Gerais, Hospital das Clínicas, Belo Horizonte MG, Brasil.; 17 Universidade Federal de São Carlos, Departamento de Medicina, Centro de Ciências Biológicas da Saúde, São Carlos SP, Brasil.

**Keywords:** Dementia, Cognitive Dysfunction, Neuropsychological Tests, Demência, Disfunção Cognitiva, Testes Neuropsicológicos

## Abstract

This consensus, performed by the Brazilian Academy of Neurology (BAN) will approach practically how to evaluate patients with cognitive complaints and how to clinically and etiologically diagnose the three clinical syndromes associated with the different stages of cognitive decline: subjective cognitive decline (SCD), mild cognitive impairment (MCI), and dementia. This BAN consensus discusses SCD diagnosis for the first time, updates MCI and dementia diagnoses, recommends the adequate cognitive tests and the relevant etiological work-up and care of patients with cognitive decline at different levels of care within the Brazilian Unified Health System. We also review the main assessment instruments used in Brazil and Latin America.

## RESUMO

Este consenso realizado pela Academia Brasileira de Neurologia (ABN) abordará de maneira prática como avaliar pacientes com queixas cognitivas e como realizar o diagnóstico clínico e etiológico das três síndromes clínicas associadas aos estágios de declínio cognitivo: declínio cognitivo subjetivo (DCS), comprometimento cognitivo leve (CCL) e demência. O diagnóstico de DCS é discutido pela primeira vez em consenso da ABN e as atualizações para o diagnóstico de CCL e demência são abordadas, bem como a recomendação para o uso de testes cognitivos apropriados, investigação etiológica pertinente e cuidados aos pacientes com declínio cognitivo nos diferentes níveis de atenção do Sistema Único de Saúde. Foi realizada pesquisa dos principais instrumentos de avaliação utilizados em nosso meio e na América Latina.

## INTRODUCTION

When we evaluate persons with cognitive complaints (self-reported or referred by an informant), the first question we must answer is whether they have dementia. Dementia is defined as a syndrome characterized by cognitive and/or behavioral decline in which symptoms interfere with activities of daily living (ADL) causing functional impairment when compared with previous functionality and that is not explained by delirium or major psychiatric^1^.

Mild cognitive impairment (MCI) is a condition in which the individual has a cognitive impairment that does not interfere with their autonomy in ADL performance. They may experience slight difficulties performing complex tasks that used to be trivial but still can maintain their independence with minimal assistance^2^. More recently, research has described a new syndrome for a group of individuals who have cognitive complaints (mainly memory) but who, when tested, show normal performance in neuropsychological tests. This situation is called subjective cognitive decline (SCD) ^(^ ^3^.

**Figure 1:**
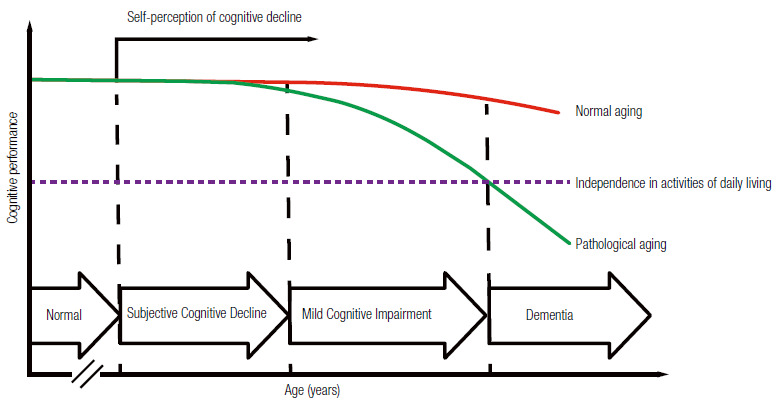
Continuum of cognitive decline in normal and pathological aging.

Therefore, dementia, MCI, and SCD are mainly clinical diagnoses. Physicians should be able to diagnose cognitive-behavioral syndromes during clinical appointments after taking patients’ medical history and performing cognition and functionality assessment. Laboratory testing and neuroimaging are helpful to define the etiology of the syndrome. Figure 1 illustrates the continuum of cognitive decline. This study aims 1) to propose a standardized assessment of patients with cognitive complaints; 2) to present the diagnostic criteria for dementia, MCI, and SCD; and 3) to propose a flowchart for investigating cognitive decline within the Brazilian Unified Health System (SUS).

## APPROACH TO PATIENTS WITH COGNITIVE AND BEHAVIORAL COMPLAINTS

### Medical history

Clinical evaluation begins with patients’ medical history. The medical history should also be taken with a family member or other close informants because anosognosia (the inability to the recognize cognitive impairment) is relatively common. Interviews with patients and their informants should be carried out separately whenever possible to address all complaints without embarrassing patients and their informant. Physicians should ask specific questions to identify which cognitive domains are affected, whether patients show behavioral symptoms, and how their cognitive decline impacts patients’ functionality. Investigable cognitive domains by medical history include memory, attention, visual-spatial functions, praxis, executive functions, and language (Table 1).

**Table 1:** Targeted medical history of patients with cognitive and behavioral complaints

**Cognitive symptoms**	Attention	Does the patient get more confused or disoriented sometimes?
		Is the patient easily distracted?
		Is the patient missing objects?
	Memory	Does the patient have more difficulties remembering recent facts than remote ones?
		Has the patient shown temporal disorientation?
		Is the patient more repetitive and asking the same questions many times?
		Does the patient need more annotations to remember appointments?
		Has the patient forgotten appointments?
	Language	Does the patient have difficulties finding words?
		Is the patient having difficulties naming objects?
		Is the patient having difficulties articulating words?
		Is the patient having difficulties understanding what you say to them?
	Spatial orientation	Has the patient got lost in previously known routes?
		Is the patient having difficulties learning new routes?
		Is the patient having difficulties locating themselves inside the house?
	Praxies	Is the patient having difficulties using utensils?
		Is the patient having difficulties dressing themselves?
	Executive functions	Is the patient having difficulties planning or organizing activities and travels?
		Is the patient having difficulties multitasking?
		Is the patient having difficulties solving daily problems?
		Is the patient having difficulties making decisions?
**Functionality**	Instrumental activities of daily living (IADLs)	Is the patient having difficulties managing their own finances?
		Is the patient having difficulties shopping?
		Is the patient having problems at work?
		Is the patient having cooking problems?
	Basic activities of daily living (BADLs)	Is the patient capable of doing the activities below independently?
		Dressing/Bathing/Hygiene/Feeding/Transference/Continence
**Behavior and neuropsychiatric symptoms**	Humor	Does the patient look sad, dejected or cry easily?
		Does the patient not see pleasure in life or says they have no future?
		Is the patient more irritable or impatient?
		Is the patient isolating themself, not getting along with others?
	Anxiety	Is the patient worried about planned events?
		Is the patient unable to relax or is excessively tense?
		Does the patient worry excessively about even trivial things?
	Apathy	Does the patient have no interest in the world around them?
		Does the patient have more difficulty engaging in conversations or tasks?
		Is the patient more indifferent?
	Disinhibition	Does the patient act impulsively, without thinking?
		Has the patient been saying things that should not be said in public?
		Has the patient acted in an embarrassing way?
		Did the patient suffer personality changes? Is the patient more socially isolated?
	Agitation	Is the patient uncooperative?
		Does the patient not allow to be helped?
		Is the patient verbally or physically aggressive?
		Does the patient repeatedly move objects around them?
		Does the patient have ritualistic or compulsive behavior?
	Delusions	Does the patient believe in things that are not real?
		Does the patient think someone is trying to harm or rob him?
		Does the patient claim that their relatives are not who they say they are?
		Does the patient claim that the house they live in is not theirs?
	Hallucinations	Does the patient report hearing voices or act as if they heard voices that are not heard by others?
		Does the patient speak to themself?
		Does the patient see people or animals that are not seen by others?
		Does the patient behave as if they saw something that others do not see?
	Appetite	Did the patient have any change in eating habits?
		Has the patient changed food preference (e.g., they started to prefer sweets)?
	Sleep	Does the patient have difficulty initiating or maintaining sleep?
		Does the patient speak or move in sleep as if they were awake?
		Does the patient often have vivid dreams or nightmares?
		Does the patient snore, wake up fatigued, or have daytime sleepiness?

During medical history, physicians should also assess conditions that can cause a false impression of cognitive impairment, such as untreated hearing or visual impairment. Furthermore, symptoms that may suggest delirium, such as fluctuation of the arousal and attention levels should be observed. Clinicians should ask patients about their use of medications, including over-the-counter drugs since many medications with anticholinergic or sedative effects can cause or worsen cognitive impairment. Moreover, clinicians should enquire about patients’ sleep pattern in search for symptoms of obstructive apnea, REM sleep behavior disorder, and daytime sleepiness. Dysautonomia symptoms such as urinary or, bowel dysfunction, erectile dysfunction and postural hypotension should also be investigated. Motor symptoms, such as changes in gait and balance, and sensory symptoms should be investigated.

### Cognitive screening tests 

Cognitive screening tests consist of brief structured instruments which enable physicians to globally assess individuals’ cognition. There are several standardized instruments, of which the Mini Mental State Examination (MMSE) is the best known and most used^4^ ^),(^ ^5^. MMSE is simple and easy to apply; the whole assessment takes from about five to seven minutes. It evaluates temporal and spatial orientation, memory, attention, calculation, language, and constructive skills. MMSE has many advantages. It is easy to apply, general practitioners know it well, and it can possibly stage disease progression. Healthy older adults keep similar MMSE scores over time, whereas patients with Alzheimer’s disease (AD), for example, lose an average of two to four points per year^6^.

Other cognitive screening tests that have been validated in Brazil are: The Blessed Information-Memory-Concentration Test^7^, Cognitive Abilities Screening Instrument - Short (CASI-S) ^(^ ^8^, the Addenbrooke’s Cognitive Examination revised version (ACE-R, which also includes the MMSE) ^(^ ^9^ ^),(^ ^10^, the Montreal Cognitive Assessment (MoCA) ^(^ ^11^ ^),(^ ^12^ and the CERAD ’s words list^13^. Among those, MoCA stands out as it evaluates executive function with the trails and clock drawing; test the visual-constructive abilities with the cube copying and clock drawing test; the episodic memory with the five words delayed recall; attention with the digit span, serials A’s and serial subtractions; language with naming, repetition, and phonemic verbal fluency; abstraction; and orientation. Therefore, the MoCA assesses a broader number of cognitive domains than the MMSE, especially the executive functions.

The MMSE and the MoCA scores are strongly affected by the level of education. We suggest using the cutoff points in Table 2 considering the several studies conducted in different Brazilian populations^5^ ^),(^ ^12^ ^),(^ ^14^ ^)-(^ ^21^. Considering the challenge of having a test to assess populations with lower educational level, Nitrini and collaborators developed the Brief Cognitive Screening Battery (BCSB) ^(^ ^22^ ^)-(^ ^24^. This test consists of naming and learning a list of 10 drawings to be freely recalled five min later, after an interference activity consisting of semantic verbal fluency (animals/1 min) and the clock drawing test^22^ ^)-(^ ^24^. It is an easy-to-apply instrument useful for diagnosing episodic memory impairment in patients with low and high educational levels^25^.

**Table 2:** Tests suggested for cognitive screening and their respective cutoff scores.

Cognitive Test	Cognitive domains	Suggested cut-off scores for the Brazilian population
Mini Mental State Examination (MMSE) ^(^ ^5^	Temporal and spatial orientation	According to education level:
	Verbal episodic memory	Illiterate: ≤19
	Attention and calculation	1-4 years: ≤24
	Language	5-8 years: ≤26
	Visual constructive skills	9-11 years: ≤27
	≥ 12 years: ≤28
Brief Cognitive Screening Battery (BCSB) ^(^ ^24^		Figure Memory Subtest:
		Incidental memory: ≤4
		Immediate memory: ≤6
		Learning: ≤6
	Visual and verbal episodic memory	Delayed recall: ≤5
	Executive functions	Recognition: ≤7
	Visual constructive skills	
		Semantic Verbal Fluency (animals) by education level^26^
		Illiterate: ≤8
		1-7 years: ≤11
		≥ 8 years: ≤12
Montreal Cognitive Assessment (MoCA) ^(^ ^12^		For Dementia (by education level):
		Illiterate: ≤8
	Executive functions	1-4 years: ≤15
	Visual constructive skills	5-8 years: ≤16
	Verbal episodic memory	9-11 years: ≤19
	Attention	≥ 12 years: ≤21
	Language	For cognitive impairment no dementia [CIND] (by education level):
	Abstraction	Illiterate: ≤11
	Temporal and spatial orientation	1-4 years: ≤17
		5-8 years: ≤19
		9-11 years: ≤19
		≥ 12 years: ≤21

The authors recommend using the MMSE and the BCSB for cognitive screening (supplementary materials). Clinicians may instead use MoCA in patients with high educational level (>12 completed years of study) and MCI or mild dementia or due to the need of better executive function assessment. Table 2 shows our suggested cut-off scores for MMSE^5^, MoCA^12^, figure delayed recall from the BCSB^24^ ^)-(^ ^26^, and the semantic fluency^27^. Eventually, dementia specialists may include other cognitive tests to assess specific cognitive domains, such as language, praxis, visuospatial skills, episodic memory, attention, and executive functions assessment batteries. Clinicians may require neuropsychological tests when cognitive screening tests are insufficiently sensitive to detect cognitive impairment, especially in individuals with MCI.

### Neurological examination

In addition to cognitive assessment, the neurological physical exam of patients with cognitive complaints must evaluate neurological signs that may point to a possible etiology. Some findings may have particular relevance, such as brisk reflexes, signs of parkinsonism, gait changes, and ocular motricity. Primitive reflexes are often seen in patients with frontal involvement (hence the term “frontalization” signs) and severe dementia. Primitive reflexes include the grasp (handgrip), rooting (searching), snout (lips protrusion), and palmomental ones.

### Functional evaluation

Daily function evaluation is a core feature in assessing patients with cognitive decline because the level of functional impairment will determine patients’ syndromes. Most instruments used for functional assessment, which are validated for Brazilian populations, are based on informants’ responses^28^. In Brazil, one of the most used instruments is Pfeffer Functional Activities Questionnaire (FAQ), which includes 10 questions especially focused on instrumental activities (supplementary material) ^(^ ^29^. It consists of a simple questionnaire that is easy to understand and quick to apply (average of seven minutes). FAQ scores range from 0 to 30 and scores above four points indicate functional impairment. This test avoids the influence of age or education level^30^ ^),(^ ^31^. Another scale which also has a Portuguese version and proved to be useful for diagnosing dementia in a Brazilian study is the Bayer activities of daily living scale (B-ADL) ^(^ ^32^. The Informant Questionnaire on Cognitive Decline in the Elderly (IQCODE) is a structured interview administered to informants which combines questions related to cognitive functioning and functional performance. It was translated and adapted for Brazilian populations and can be helpful to screen dementia in individuals with varying educational level^33^ ^),(^ ^34^. The Katz scale, which has been translated and adapted for Brazilian populations, can be used to assess basic activities of daily living (BADLs) ^(^ ^28^ ^),(^ ^35^.

### Cognitive impairment staging

When a person is diagnosed with cognitive decline, clinicians need to stage this condition. The Clinical Dementia Rating (CDR) scale has been used worldwide for staging cognitive decline and has been validated for Brazil^36^. CDR rates six domains: memory, orientation, judgment and problem solving, community affairs, home and hobbies, and personal care^37^. Clinicians can use a semi-structured interview to assist with scoring. The first three domains are assessed with patients and informants and the last three domains, with informants only. The CDR global score ranges between 0 (normal), 0.5 (MCI), 1 (mild dementia), 2 (moderate dementia), and 3 (severe dementia).

A brief cognitive assessment using the MMSE and dementia staging by the CDR are needed to request the Brazilian Unified Health System (SUS) to provide the symptomatic pharmacological treatment for dementia due to AD. SUS provides acetylcholinesterase inhibitors for mild to moderate AD and memantine to moderate to severe AD^38^. This consensus will comprehensively discuss dementia treatment^39^.

## SUBJECTIVE COGNITIVE DECLINE (SCD) 

### Concept and diagnostic criteria

Subjective cognitive decline (SCD) is defined as self-perceived cognitive decline with neither objective impairment on cognitive tests nor functional impairment on ADLs^40^ ^),(^ ^41^. The literature has used several expressions to define self-perceived cognitive impairment, such as “subjective memory complaint,” “subjective cognitive impairment,” and “subjective cognitive complaint” ^(^ ^3^ ^),(^ ^42^. In 2014, an international working group (the Subjective Cognitive Decline Initiative, SCD-I) proposed diagnostic criteria for SCD focused on standardizing terminology for research in preclinical AD (Table 3). This group recommends to use the term “cognitive,” instead of “memory,” because the first symptoms may not be limited only to amnestic and “decline” instead of a “complaint,” as it refers to the idea of progressive deterioration and not only an isolated and non-progressive complaint.

**Table 3:** Approaches to Classifying SCD among a sample of Subjective Cognitive Decline Initiative (SCD-I) Working Group studies^3,42^.

Criteria 1 and 2 must be present:
**1.** Self-experienced persistent decline in cognitive capacity in comparison with a previously normal status, unrelated to an acute event.
**2.** Normal performance on standardized cognitive tests, which are used to classify mild cognitive impairment (MCI) (adjusted for age, gender, and education level).
**Exclusion criteria:**
**1.** Diagnosis of mild cognitive impairment or dementia.
**2.** Cannot be explained by a psychiatric* or neurologic disease (apart from AD), medical disorder, medication, or substance use.

*Symptoms of depression or anxiety that fail to meet criteria for a psychiatric disorder are not considered exclusion criteria.

### Epidemiology

A Mayo Clinic study showed an SCD prevalence between 12.3% and 57% among cognitively healthy participants aged between 70 and 95 years. Overall, 24% of the total population reported memory concerns^43^. Similarly, a Brazilian study, conducted in the municipality of Tremembé, reported an SCD prevalence of 27.6% among all participants over 60 years old, which represented 45.2% of individuals with normal cognitive tests^44^. Longitudinal epidemiological studies have associated SCD with an increased risk for progression to MCI and dementia^3^ ^),(^ ^43^ ^),(^ ^45^ ^),(^ ^46^. In a meta-analysis of 29 studies, the annual conversion rate from SCD to MCI and dementia was around 6.7% and 2.3%, respectively, compared with a conversion to dementia of only 1% among older adults without SCD. Among studies which conducted follow-ups for longer than four years, the evolution to MCI and dementia reached 26.7% and 14.1%, respectively^47^. Furthermore, older adults with SCD showed a higher prevalence of positive AD biomarkers^48^ ^)-(^ ^51^.

### SCD etiologies

Many people with SCD remain stable or even improve. This is because not only neurodegenerative diseases (such as AD) are among the causes of SCD. Several other conditions can be associated with SCD, such as normal aging, personality traits, psychiatric disorders (especially depression and anxiety), sleep disorders (like obstructive sleep apnea syndrome), and the use of psychoactive medications with anticholinergic or GABAergic effects^42^ ^),(^ ^52^.

Therefore, several questions arise: How to identify which individuals within this heterogeneous group will progress to MCI or dementia? What are the SCD characteristics which increase the risk for progression? In other words, how can we determine whether an individual with SCD has an underlying preclinical AD?

Therefore, some authors have proposed the term “SCD plus.” Patients with SCD plus have a higher probability of showing a neurodegenerative pathology. Individuals at increased risk of conversion to Alzheimer-type dementia are older adults (≥ 60 years) with progressive amnestic cognitive complaints within the last five years who worry about this decline and has family members confirming this decline^3^ ^),(^ ^40^.

It is important to identify people with SCD at higher risk of developing AD mainly in the context of therapeutic clinical trials. In clinical practice, patients with SCD must be followed up, especially those who meet the SCD plus criteria. Although there are no pharmacological treatments, non-pharmacological interventions, should be encouraged such as aerobic exercise and control of cardiovascular risk factors. Patients with SCD should be screened for psychiatric disorders, such as depression, sleep disorders like sleep apnea, as well as for the use of anticholinergic medications and other clinical disorders associated with cognitive impairment, such as hypothyroidism.

## MILD COGNITIVE IMPAIRMENT (MCI) 

### Concept and diagnostic criteria

MCI consists of an intermediate clinical condition between normal aging and dementia, which SCD may precede. The MCI criteria initially defined by Petersen emphasized the presence of complaints and memory impairment with performance on tests usually 1.5 standard deviations below the mean for age and education, during basic activities of daily living^53^. The literature has described the risk of progression to AD as 10 to 12% per year^53^. Thereafter, research has recognized MCI as a more heterogeneous and comprehensive entity regarding its clinical presentation, etiology, and prognosis, considering deficits in other cognitive domains besides memory^54^ ^),(^ ^55^. The MCI definition includes the following criteria: (1) cognitive complaints reported by the patient and/or informant; (2) report of cognitive decline during the past year; (3) changes in cognition (memory and/or other domains) evinced at the clinical objective evaluation when compared with normal adults of the same age and educational level; (4) no difficulty with daily activities, preserved general cognitive functioning; and (5) absence of dementia^56^ ^),(^ ^57^.

**Figure 2:**
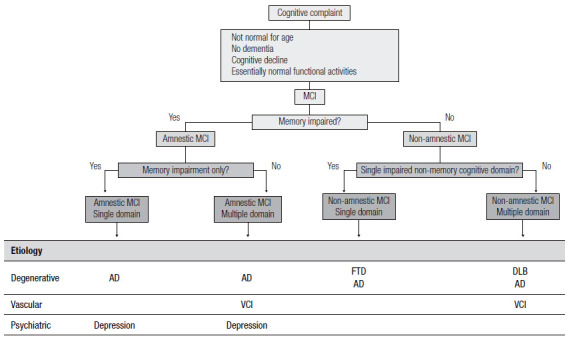
Diagnostic classification of MCI after the Key Symposium criteria. MCI: Mild Cognitive Impairment; AD: Alzheimer’s disease; VCI: vascular cognitive impairment; FTD: frontotemporal dementia; DLB: dementia with Lewy bodies.

Over the years, these clinical criteria received updates since the MCI definition initially proposed in 1999 (known as the Mayo Clinic criteria^53^) had gaps, such as involvement of other cognitive domains, the possibility that cognitive complaints could come from informants, and acceptance of minimally impaired complex instrumental functions. These updates were included in the 2004 Key Symposium consensus^55^. From then on, MCI includes four subtypes: single-domain amnestic MCI, multiple-domain amnestic MCI, single-domain non-amnestic MCI, and multiple-domain non-amnestic MCI (Figure 2). The several MCI subtypes are due to possible degenerative, vascular, metabolic, and psychiatric etiologies, among others^55^.

In 2011, the National Institute on Aging and Alzheimer’s Association (NIA-AA) again updated the consensus for the clinical diagnosis of MCI, establishing diagnostic criteria for the symptomatic stage of AD pre-dementia^58^, thus creating the AD-due MCI terminology for cases with the clinical characteristics, as per Table 4. Moreover, it included the presence of biomarkers into the criteria for research, specialized centers, and clinical trials, allowing for different degrees of AD etiology probability^58^. Finally, in 2013, the MCI consensus underwent yet another terminology increments during the establishment of the Diagnostic and Statistical Manual of Mental Disorders, Fifth Edition (DSM-5), which recognized it as a mild neurocognitive disorder^59^. In 2022, the 11th edition of the International Statistical Classification of Diseases and Related Health Problems (ICD 11) proposed the term mild neurocognitive disorder^60^.

**Table 4:** Diagnostic criteria for Mild Cognitive Impairment due to Alzheimer’s disease by the National Institute on Aging and Alzheimer’s Association (NIA-AA)^58^.

Clinical and cognitive characteristics
Cognitive worries reflecting a change in cognition reported by patients, informants or clinicians (i.e., historical or observed evidence of decline over time)
Objective evidence of impairment in one or more cognitive domains, typically including memory (i.e., formal, or bedside testing to establish level of cognitive function in multiple domains)
Preservation of independence in functional abilities. They may show slight problems performing complex tasks, such as taking longer to complete the task or making some mistakes
Not demented
**Etiology of MCI consistent with AD pathophysiological process**
Rule out vascular, traumatic, medical causes of cognitive decline, where possible
Provide evidence of longitudinal decline in cognition, when feasible
Report history consistent with AD genetic factors

MCI: Mild Cognitive Impairment; AD: Alzheimer’s disease.

For the clinical diagnosis, in addition to a detailed medical history, it is important to question about the presence of systemic diseases, use of medications and have the evidence of objective cognitive assessment with a mild decline in relation to the individuals’ previous cognitive level in one or more cognitive domains (complex attention, executive function, learning and memory, language, perceptuomotor, or social cognition), besides not meeting the diagnostic criteria for dementia^58^. Therefore, cognitive deficits in MCI do not interfere with the ability to be independent in ADLs, thus complex instrumental activities of daily life are preserved, such as paying bills or controlling medications, but there may be need for more effort or compensatory strategies^58^. Independence in instrumental ADLs distinguishes MCI from dementia. Nevertheless, when compared to normal aging, individuals with MCI perform more poorly^59^.

### Epidemiology

Epidemiological studies of MCI, although scarce and heterogeneous regarding their adopted criteria (used tests and educational level of the studied population), are important since longitudinal studies have shown that people with MCI are at increased risk of developing dementia, up to five times as high annually than the general population^62^ ^)-(^ ^65^. The estimated prevalence of MCI in most international population studies ranges from 10 to 22% in people aged 65 years or older^62^ ^),(^ ^64^ ^)-(^ ^68^. A meta-analysis found a wide range of definitions and concepts to estimate MCI prevalence and incidence, representing a challenge for researchers’ understanding of the burden of this disease; MCI prevalence and incidence rates ranged, respectively, from 3 to 42% and from 21.5 to 71.3 per 1000 person-years, and the prevalence of cognitive impairment with no dementia (CIND) ranged from 5.1 to 35.9%^69^.

We find varying terminology in the few population studies conducted in Brazil. Overall, two studies, conducted in the municipality of Porto Alegre (Rio Grande do Sul State), found an MCI prevalence of 6.1%^70^ and a 13.2 per 1000 person-years incidence^71^, whereas the study conducted in the municipality of Tremembé, in São Paulo State, found a CIND prevalence of 19.5% for the population aged 60 years and older^72^. 

## DEMENTIA: A SYNDROMIC APPROACH

### Concept and diagnostic criteria

The criteria for dementia proposed in 2011 by the NIA-AA and the Brazilian Academy of Neurology pare shown in Table 5 ^1^ ^),(^ ^71^. In 2014, the American Psychiatric Association through the DSM-5, proposed the use of the term major neurocognitive disorder and expanded the diagnosis of dementia to situations where there is only one cognitive domain affected^58^.

**Table 5:** Diagnostic criteria for dementia according to the National Institute on Aging and Alzheimer’s Association (NIA-AA) and the Brazilian Academy of Neurology^1^ ^),(^ ^73^.

1. Dementia is diagnosed when there are cognitive or behavioral (neuropsychiatric) symptoms that:
1.1. Interfere with the ability to work or to carry out usual activities
1.2. Represent a decline from previous levels of functioning and performance;
1.3. Cannot be explained by delirium or major psychiatric disorder.
2. Cognitive impairment is detected and diagnosed by a combination of:
2.1. Medical history with the patient and a reliable informant; and
2.2. An objective cognitive assessment, either a “bedside” mental status examination or neuropsychological testing. Neuropsychological testing should be performed when medical history and bedside mental status examination cannot provide a confident diagnosis.
3. Cognitive or behavioral impairment involves a minimum of two of the following domains:
3.1. Memory: impaired ability to acquire and remember new information - symptoms include repetitive questions or conversations, misplacing personal belongings, forgetting events or appointments, getting lost on a familiar route;
3.2. Executive functions: impaired reasoning and carrying out complex tasks and judging - symptoms include poor understanding of safety risks, inability to manage finances, poor decision-making ability, inability to plan complex or sequential activities;
3.3. Visuospatial abilities: symptoms include inability to recognize faces or common objects or to find objects in the visual field, inability to handle utensils and dressing oneself for reasons other than visual or motor deficiency.
3.4. Language (speaking, reading, writing): symptoms include difficulty in finding common words while speaking, hesitations; speech, spelling, and writing errors and exchange of words or phonemes, unexplicable by a sensory or motor deficit.
3.5. Behavior or personality: symptoms include uncharacteristic mood fluctuations such as agitation, impaired motivation, initiative, apathy, loss of drive, social withdrawal, decreased interest in previous activities, loss of empathy, compulsive or obsessive behaviors, and socially unacceptable behaviors.

For the most recent edition of the International Statistical Classification of Diseases and Related Health Problems (ICD-11), published in 2019 and which will come into force in 2022, there is still a need for cognitive decline in at least two cognitive domains for the diagnosis of dementia , not allowing the diagnosis of cognitive decline in a single domain^72^ ^),(^ ^73^.

## Epidemiology

The prevalence of dementia in Brazil ranges from 5.1 to 17.5%, varying according to the studied region and study design^75^. The prevalence of dementia in the world population aged ≥60 years is 5 to 7% in most regions, with the highest prevalence in Latin America, at 8.5%^76^. In a Brazilian study, the prevalence of dementia is even higher, reaching 23% in illiterate individuals aged 60 years or older living at the municipality of Tremembé^72^.

In Brazil, a study suggests that 32.3% of dementia cases are due to seven modifiable risk factors: diabetes mellitus, systemic arterial hypertension, middle-age obesity, physical inactivity, depression, smoking, and poor education^77^. Reducing the prevalence of each risk factor by 10% or 20% per decade could potentially reduce the prevalence of dementia in 2050 by 8.7 or 16.2% in Brazil^77^.

## Etiologies of MCI and dementia

**Figure 3:**
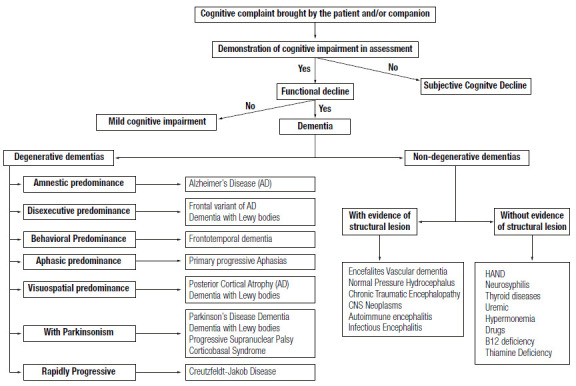
Main etiological differential diagnoses of dementia.

The literature divides the etiologies of dementias into primary (or neurodegenerative) and secondary ones (Figure 3). The altered accumulation of misfolded proteins (misfolded proteins diseases) in the central nervous system (CNS) ^(^ ^78^ ^)^ pathologically characterizes degenerative dementias. AD, Lewy body (LBD), and frontotemporal dementias (FTD) are the most frequent types of primary dementias. Other various neurological diseases, usually associated with movement disorders (such as parkinsonism or chorea), can develop dementia throughout its evolution, such as Parkinson’s disease, progressive supranuclear palsy, corticobasal syndrome, and Huntington’s disease. 

Epidemiological and clinical studies found that AD dementia is the most prevalent cause of dementia in Brazil, followed by vascular dementia (VD) due to cerebrovascular disease^75^. A study conducted with clinicopathological analysis also found the same ordering of causes related to dementia^79^. Another study from a Brazilian brain bank with 480 participants found that 50% of its sample met the neuropathological criteria for AD, 35%, VD; 18%, LBD; 17% received other diagnoses (e.g., frontotemporal lobar degeneration); and 20% showed mixed pathology (most were AD associated with VD) ^(^ ^80^.

**Figure 4:**
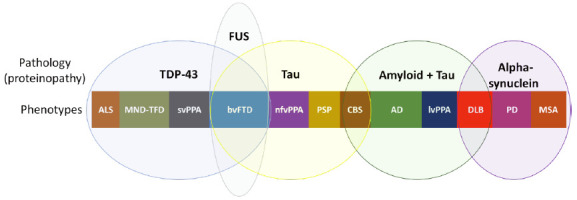
Spectrum of proteinopathies causing neurodegenerative dementias and their respective clinical phenotypes. Pathological proteins: TDP-43, FUS, Tau, beta-amyloid and alpha-synuclein. FTD: Frontotemporal dementia; ALS: Amyotrophic lateral sclerosis; MND: Motor neuron disease; svPPA: Semantic variant of primary progressive aphasia; nfvPPA: Nonfluent (or agrammatic) variant of primary progressive aphasia; CBS: Corticobasal syndrome; PSP: Progressive supranuclear palsy; AD: Alzheimer’s disease; lvPPA: Logopenic variant of primary progressive aphasia; DLB: Dementia with Lewy bodies; PD: Parkinson’s disease; MSA: Multiple systems atrophy.

Historically, neurodegenerative dementias were described based on their clinical phenotypes (e.g., AD is described as amnestic dementia in most cases). However, in the last two decades, studies searching an early diagnosis based on biomarkers , that allow the identification of pathological proteins *in vivo* have been increasing^77^. Interestingly, the same pathological protein can lead to different phenotypes, while the same phenotype can be associated with different proteins^80^. Figure 4 illustrates this broad spectrum of proteinopathies and clinical phenotypes. 

Secondary causes are potentially treatable and should be investigated for early treatment. The most frequent cause of secondary dementia is VD, which is the second leading cause of dementia in Brazil. The investigation of risk factors and mechanisms of cerebral vascular involvement is essential for adequate treatment of VD patients^78^. Vitamin B12 deficiency, neurosyphilis, and normal pressure hydrocephalus are causes of secondary dementia that should be initially ruled out.

Rarer causes, shown as rapidly progressive conditions (evolving to dementia within one to two years of the onset of the first symptoms, usually within weeks to months) are prion, autoimmune, and infectious dementias, among others^82^. Rapidly progressive dementia (RPD) requires extensive investigation, which must be carried out early to avoid cognitive sequelae in cases of potentially reversible etiology.

Depression is one of the main differential diagnoses of dementia. Major depressive disorder courses with cognitive decline, executive dysfunction, and attention deficit^83^. The relation between dementia and depression is complex because depressive symptoms can be the initial manifestation of dementia. Therefore, depression is a risk factor for dementia and severe depression can cause potentially reversible dementia^84^. Depression is one of the 12 modifiable risk factors responsible for 40% of dementia cases worldwide^85^. Accordingly, every patient with depressive symptoms should receive adequate treatment for depression. However, if cognitive deficits remain after optimized treatment, clinical practice should suspect of a degenerative etiology.

Cognitive decline associated with HIV infection, called HAND (HIV-associated neurocognitive disorder), shows an increasing prevalence after the use of highly effective antiretroviral treatment for people living with HIV^86^. HAND prevalence in Brazil ranges from 52.4% to 73.6%, with a higher percentage of asymptomatic cases, named asymptomatic neurocognitive impairment^87^ ^),(^ ^88^.

Research should investigate the use of psychoactive medications, especially benzodiazepines, antipsychotics, non-benzodiazepine hypnotics, and anticholinergic medications, as possible etiologies of dementia. Table 6 shows some anticholinergic medications associated with cognitive decline^89^.

**Table 6:** List of drugs with centrally acting strong anticholinergic properties*.

**Antiarrhythmic** Disopyramide	**Antimuscarinics** (urinary incontinence) Darifenacin Fesoterodine Flavoxate Oxybutynin Solifenacin Tolterodine Trospium
**Antidepressants** Amitriptyline Amoxapine Desipramine Desipramina Doxepin (>6mg) Imipramine Nortriptyline Paroxetine Protriptyline Trimipramine	
	**Antiparkinsonian agents** Biperiden Trihexyphenidyl Benztropine
**Antiemetics** Prochlorperazine Promethazine	**Antipsychotics** Chlorpromazine Clozapine Loxapine Olanzapine Perphenazine Thioridazine Trifluoperazine

**Antihistamines** (first generation) Brompheniramine Carbinoxamine Chlorpheniramine Clemastine Cyproheptadine Dexbrompheniramine Dexchlorpheniramine Dimenhydrinate Diphenhydramine (oral) Doxylamine Hydroxyzine Meclizine Clidinium-chlordiazepoxide Dicyclomine Homatropine (excludes ophthalmic) Hyoscyamine Methscopolamine Propantheline Promethazine Pyrilamine Triprolidine	
	**Antispasmodics** Atropine (excludes ophthalmic) Belladonna alkaloids Scopolamine (excludes ophthalmic)
	**Skeletal muscle relaxants** Cyclobenzaprine Orphenadrine

* Adapted from Beers Criteria^89^.

### Etiological investigation of MCI and dementia

The current recommendation for investigating MCI and dementia in the Brazilian population involves a neuroimaging study (computed tomography scan - CT - or, ideally, brain magnetic resonance imaging - MRI) and laboratory tests which investigate non-neurodegenerative etiologies. These tests include complete blood count, serum concentrations of creatinine, TSH, albumin, liver enzymes, vitamin B12, ionized calcium, serological reactions for syphilis; and HIV serology for those with atypical clinical presentations or suggestive symptoms^90^. Clinical practice should also request cerebrospinal fluid (CSF) examination for patients with early-onset dementia (before 65 years of age), atypical presentations, and suspected inflammatory, prion, or infectious diseases^90^. Clinicians should always assess RPD with brain MRIs (with diffusion-weighted imaging), electroencephalogram, CSF, and broader laboratory investigation, depending on the clinical hypotheses.

This MRI recommendation is especially due to the possibility of finding atrophy in the hippocampal region, which is impossible by CT scan in the early stages of the disease. The finding of atrophy in the mesial temporal regions is suggestive of the diagnosis of MCI due to AD, however, it is not exclusive to this pathology, since a considerable proportion of cases of amnestic MCI with hippocampal atrophy may have different causes other than AD, denominated SNAP (suspected non-Alzheimer pathophysiology) ^(^ ^91^. Another advantage of brain MRI is its better identification of cerebrovascular disease, especially of small vessel disease.

The clinical indications for biomarkers include 1) identification of individuals with MCI and mild dementia due to AD pathology and 2) diagnostic uncertainty about the dementia etiology. Biomarkers available for clinical use can be divided into specific AD pathology or neurodegeneration biomarkers. Specific AD pathology biomarkers include 1) measurement of the beta-amyloid peptide and phosphorylated tau (phospho-tau) in the CSF and 2) positron emission computed tomography (PET) with amyloid peptide marker and PET with marker for tau protein. Until now, PET-amyloid is offered in few centers in Brazil and PET-tau is still unavailable in Brazil. Neurodegeneration biomarkers consist of PET with 18F-fluorodeoxyglucose (PET-FDG), measurement of total tau protein in the CSF, and light chain neurofilament. More recently, plasma biomarkers have been developed but they still lack validation for clinical use (tau-181, tau-217) ^(^ ^92^ ^)-(^ ^94^.

Confirmation of the AD *in vivo* requires amyloid and tau pathology. CSF measurement or PET-amyloid uptake can confirm the amyloid pathology, whereas measuring CSF phospho-tau or PET-tau uptake can confirm the Tau pathology. PET-FDG is a neurodegeneration biomarker whose metabolic pattern may suggest the pathology of degenerative dementias with good sensitivity and specificity (e.g., the pattern of hypometabolism in the areas of temporoparietal association, posterior cingulate, and precuneus has a sensitivity and specificity >90% for AD diagnosis) ^(^ ^95^. Furthermore, PET FDG is used in the diagnostic criteria of LBD, primary progressive aphasias, and FTD.

## PROPOSAL FOR THE MANAGEMENT OF PATIENTS WITH COGNITIVE DECLINE AT DIFFERENT LEVELS OF CARE IN THE BRAZILIAN PUBLIC HEALTH SERVICE - SUS

### Primary healthcare

Identification of modifiable risk factors for dementia prevention should be done at primary health care. Recently, a study described 12 important modifiable risk factors to prevent or delay the onset of 40% of dementia in the world and 56% in low- and middle-income countries^84^. They are diagnosed at different stages of life, with the possibility of involvement by the health system in all of them. Risk factors include: 1) age up to 45 years: low educational level; 2) age from 45 to 65 years old: systemic arterial hypertension, obesity, hearing loss, traumatic brain injury, and alcohol abuse; and 3) age above 65 years old: smoking, depression, sedentary lifestyle, diabetes, social isolation, and air pollution. Clinical practice must consider preventive and educational actions to identify, promote treatment, and combat these different factors^84^.

Primary care physicians should actively seek out complaints of cognitive decline in patients, especially after 60 years of age. Clinicians should perform screening tests for cognitive and functional decline in patients with cognitive complaints (or reported by caregivers). In case of a clinical diagnosis of MCI or dementia, physicians should request etiological work-up. When ruling out potentially reversible causes, patients should be referred to secondary healthcare, to neurology, geriatrics, or psychiatry specialists, with a hypothesis of degenerative or vascular etiology. The RPD cases must be promptly referred to secondary or tertiary care, as a matter of urgency.

Primary care physicians must receive back patients referred to secondary or tertiary care for long-term follow-up of AD and VD dementia. Primary care physicians should request a new specialist evaluation in case of any doubt regarding the follow-up of the case.

### Secondary healthcare

Physicians in secondary care emergencies must be able to identify cases of RPD, because patients often seek emergency units due to the rapid progression of symptoms. When recognizing a case of RPD, physicians must urgently conduct an initial investigation and, preferably, refer patients to tertiary care for extensive work-up.

Specialists (neurologists, geriatricians, and psychiatrists) who receive patients referred for dementia, whose secondary etiologies were excluded at the primary level, should confirm the diagnosis and establish treatment. For cases in which there is no greater complexity in managing neuropsychiatric symptoms, recommendations on how to treat dementia should be explained to the primary care physician who will follow up the patient. 

Secondary care should request biomarker tests when necessary. Physicians should request biomarker tests in atypical cases (initial non-amnestic clinical presentation or early-onset dementia) and interpreting them requires trained secondary care professionals. If it is impossible to assess biomarkers or the professional is unfamiliar with result interpretation, patients must be referred to tertiary centers.

In cases of early-onset dementia in which the specialist demands further etiological investigation, patients should be referred to tertiary services for diagnosis elucidation.

### Tertiary healthcare

Cases which maybe referred to tertiary care include RPD, early-onset dementia, suspected genetic form of cognitive decline, cases of unclear diagnosis, cases of difficult management of neuropsychiatric symptoms, and cases for biomarker investigation which secondary care is unable to assess.

**Figure 5:**
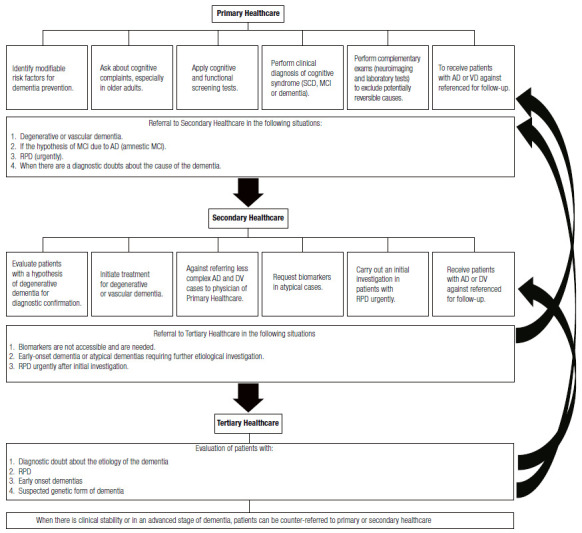
Hierarchy of care for patients with cognitive syndromes according to the levels of care in the Brazilian Unified Health System (SUS). AD: Alzheimer’s Disease, VD: Vascular Dementia, MCI: Mild cognitive impairment; SCD: Subjective cognitive decline; RPD: Rapidly progressive dementia.

In cases of clinical stability or advanced stage of dementia, patients can be counter-referred to primary or secondary care. Figure 5 summarizes the competences of each level of healthcare in caring for patients with cognitive decline.

## Supplementary Material I

**BRIEF COGNITIVE SCREENING BATTERY** ^1^

Available at: https://www.demneuropsy.com.br/imageBank/suplementar/Brief_Cognitive_Screening_Battery.pdf

**REFERENCES**

1. Nitrini R, Brucki SMD, Yassuda MS, Fichman HC, Caramelli P. The Figure Memory Test: diagnosis of memory impairment in populations with heterogeneous educational background. Dement Neuropsychol. 2021 Apr-Jun;15(2):173-185. doi: 10.1590/1980-57642021dn15-020004.

## Supplementary Material II

**FUNCTIONAL ACTIVITIES QUESTIONNAIRE** ^3^

This test, created by Pfeffer et al.^1^, was modified to be applied in people with heterogeneous educational attainment by Amaducci et al^2^. in 1991 for a worldwide epidemiological study organized by the WHO and then by Nitrini et al., in 1997^3^ for an epidemiological study in Brazil^4^ so it could be answered directly by the informant. However, if necessary, the examiner can apply the questionnaire. Answers always follow the same criteria. Ask the informant to circle the correct answer.

**Table:**

Patient Name:		_______________________________________________________________	
Date:		____/____/____	
Informant(s):	________________________	(degree of kinship or function):	_________________________
1) Do they handle their own money?			
0 = Normal		0 = Never did, but could do it now	
1 = Does, with difficulty		1 = Never did it and now would have difficulty	
2 = Needs help			
3 = Unable			
2) Can they buy clothes, food, objects for their home by themselves?			
0 = Normal		0 = Never did, but could do it now	
1 = Does, with difficulty		1 = Never did it and now would have difficulty	
2 = Needs help			
3 = Unable			
3) Can they heat water to make coffee and turn off the stove?			
0 = Normal		0 = Never did, but could do it now	
1 = Does, with difficulty		1 = Never did it and now would have difficulty	
2 = Needs help			
3 = Unable			
4) Can they prepare a meal?			
0 = Normal		0 = Never did, but could do it now	
1 = Does, with difficulty		1 = Never did it and now would have difficulty	
2 = Needs help			
3 = Unable			
5) Can they keep up with current events or community or neighborhood ones?			
0 = Normal		0 = Never did, but could do it now	
1 = Does, with difficulty		1 = Never did it and now would have difficulty	
2 = Needs help			
3 = Unable			
6) Can they pay attention, understand, and discuss a radio or television show or newspaper and magazine news?			
0 = Normal		0 = Never did, but could do it now	
1 = Does, with difficulty		1 = Never did it and now would have difficulty	
2 = Needs help			
3 = Unable			
7) Can they remember commitments, family events, and holidays?			
0 = Normal		0 = Never did, but could do it now	
1 = Does, with difficulty		1 = Never did it and now would have difficulty	
2 = Needs help			
3 = Unable			
8) Can they handle their own medications?			
0 = Normal		0 = Never did, but could do it now	
1 = Does, with difficulty		1 = Never did it and now would have difficulty	
2 = Needs help			
3 = Unable			
9) Can they walk around the neighborhood and find their way back home?			
0 = Normal		0 = Never did, but could do it now	
1 = Does, with difficulty		1 = Never did it and now would have difficulty	
2 = Needs help			
3 = Unable			
10) Can they be safely left at home alone?			
0 = Normal		0 = Never stayed, but could stay now	
1 = Yes, but with precautions		1 = Never stayed and now would have difficulty	
2 = Yes, for short periods			
3 = No			

**Score (0 to 30) = ____**

**Scores higher than 5 indicate functional impairment**

**REFERENCES**

1. Pfeffer RI, Kurosaki TT, Harrah CH, Chance JM, Filos S. Measurement of functional activities in older adults in the community. J Gerontol. 1982;37(3):323-9. doi:10.1093/geronj/37.3.323.

2. Amaducci L, Baldereschi M, Amato MP, Lippi A, Nencini P, Maggi S, Litvak J. The World Health Organization cross-national research program on age-associated dementias. Aging (Milano). 1991;3(1):89-96. doi:10.1007/BF03323983.

3. Herrera E Jr, Caramelli P, Nitrini R. Estudo epidemiológico populacional de demência na cidade de Catanduva, estado de São Paulo, Brasil. Rev Psiquiatr Clín. 1997;25(2),70-3.

4. Herrera E Jr, Caramelli P, Silveira AS, Nitrini R. Epidemiologic survey of dementia in a community-dwelling Brazilian population. Alzheimer Dis Assoc Disord. 2002;16(2):103-8. doi:10.1097/00002093-200204000-00007.

## References

[1] McKhann GM, Knopman DS, Chertkow H, Hyman BT, Jack CR, Kawas CH (2011). The diagnosis of dementia due to Alzheimer's disease: recommendations from the National Institute on Aging-Alzheimer's Association workgroups on diagnostic guidelines for Alzheimer's disease. Alzheimers Dement.

[2] Petersen RC (2011). Clinical practice Mild cognitive impairment. N Engl J Med.

[3] Jessen F, Amariglio RE, Van Boxtel M, Breteler M, Ceccaldi M, Chételat G (2014). A conceptual framework for research on subjective cognitive decline in preclinical Alzheimer's disease. Alzheimers Dement.

[4] Folstein MF, Folstein SE, McHugh PR (1975). "Mini-mental state" A practical method for grading the cognitive state of patients for the clinician. J Psychiatr Res.

[5] Brucki SMD, Nitrini R, Caramelli P, Bertolucci PHF, Okamoto IH (2003). Sugestões para o uso do mini-exame do estado mental no Brasil. Arq Neuropsiquiatr.

[6] Barocco F, Spallazzi M, Concari L, Gardini S, Pelosi A, Caffarra P (2017). The Progression of Alzheimer's Disease: Are Fast Decliners Really Fast? A Four-Year Follow-Up. J Alzheimers Dis.

[7] Viana GS, Rouquayrol MZ, Bruin VM, Albuquerque JJ (1991). Use of the Information, Memory and Concentration (IMC) Test in the epidemiological study of senile dementia in Fortaleza,Ceará (Brazil). Cad Saude Publica.

[8] Damasceno A, Delicio AM, Mazo DFC, Zullo JFD, Scherer P, Ng RTY (2005). Validation of the Brazilian version of mini-test CASI-S. Arq Neuropsiquiatr.

[9] Carvalho VA, Caramelli P (2007). Brazilian adaptation of the Addenbrooke's Cognitive Examination-Revised (ACE-R). Dement Neuropsychol.

[10] César KG, Yassuda MS, Porto FHG, Brucki SMD, Nitrini R (2017). Addenbrooke's cognitive examination-revised: Normative and accuracy data for seniors with heterogeneous educational level in Brazil. Int Psychogeriatrics.

[11] Nasreddine ZS, Phillips NA, Bédirian V, Charbonneau S, Whitehead V, Collin I (2005). The Montreal Cognitive Assessment, MoCA: a brief screening tool for mild cognitive impairment. J Am Geriatr Soc.

[12] Cesar KG, Yassuda MS, Porto FHG, Brucki SMD, Nitrini R (2019). MoCA Test normative and diagnostic accuracy data for seniors with heterogeneous educational levels in Brazil. Arq Neuropsiquiatr.

[13] Bertolucci PH, Okamoto IH, Brucki SM, Siviero MO, Toniolo J, Ramos LR (2001). Applicability of the CERAD neuropsychological battery to Brazilian elderly. Arq Neuropsiquiatr.

[14] Bertolucci PH, Brucki SM, Campacci SR, Juliano Y (1994). The Mini-Mental State Examination in a general population impact of educational status. Arq Neuropsiquiatr.

[15] Almeida OP (1998). Mini mental state examination and the diagnosis of dementia in Brazil. Arq Neuropsiquiatr.

[16] Laks J, Batista EMR, Guilherme ERL, Contino ALB, Faria MEV, Figueira I (2003). Mini-mental state examination in community- dwelling elderly preliminary data from Santo Antônio de Pádua, Rio de Janeiro, Brazil. Arq Neuropsiquiatr.

[17] Castro-Costa E, Fuzikawa C, Uchoa E, Firmo JOA, Lima-Costa MF (2008). Norms for the mini-mental state examination: adjustment of the cut-off point in population-based studies (evidences from the Bambuí health aging study). Arq Neuropsiquiatr.

[18] Kochhann R, Varela JS, Lisboa CSM, Chaves MLF (2010). The Mini Mental State Examination: Review of cutoff points adjusted for schooling in a large Southern Brazilian sample. Dement Neuropsychol.

[19] Memória CM, Yassuda MS, Nakano EY, Forlenza OV (2013). Brief screening for mild cognitive impairment: validation of the Brazilian version of the Montreal cognitive assessment. Int J Geriatr Psychiatry.

[20] Apolinario D, Santos MF, Sassaki E, Pegoraro F, Pedrini AVA, Cestari B (2018). Normative data for the Montreal Cognitive Assessment (MoCA) and the Memory Index Score (MoCA-MIS) in Brazil: Adjusting the nonlinear effects of education with fractional polynomials. Int J Geriatr Psychiatry.

[21] Lourenço RA, Veras RP (2006). Mini-Mental State Examination: psychometric characteristics in elderly outpatients. Rev Saude Publica.

[22] Nitrini R, Lefèvre BH, Mathias SC, Caramelli P, Carrilho PE, Sauaia N (1994). Neuropsychological tests of simple application for diagnosing dementia. Arq Neuropsiquiatr.

[23] Nitrini R, Caramelli P, Porto CS, Charchat-Fichman H, Formigoni AP, Carthery-Goulart MT (2007). Brief cognitive battery in the diagnosis of mild Alzheimer ' s disease in subjects with medium and high levels of education. Dement Neuropsychol.

[24] Nitrini R, Bucki SMD, Yassuda MS, Fichman HC, Caramelli P (2021). The Figure Memory Test: diagnosis of memory impairment in populations with heterogeneous educational background. Dement Neuropsychol.

[25] Nitrini R, Caramelli P, Herrera E, Porto CS, Charchat-Fichman H, Carthery MT (2004). Performance of illiterate and literate nondemented elderly subjects in two tests of long-term memory. J Int Neuropsychol Soc.

[26] Yassuda MS, Silva HS, Lima-Silva TB, Cachioni M, Falcão DVS, Lopes A (2017). Normative data for the Brief Cognitive Screening Battery stratified by age and education. Dement Neuropsychol.

[27] Caramelli P, Carthery-Goulart MT, Porto CS, Charchat-Fichman H, Nitrini R (2007). Category fluency as a screening test for Alzheimer disease in illiterate and literate patients. Alzheimer Dis Assoc Disord.

[28] Chaves MLF, Godinho CC, Porto CS, Mansur L, Carthery-Goulart MT, Yassuda MS (2011). Cognitive, functional and behavioral assessment: Alzheimer's disease. Dement Neuropsychol.

[29] Pfeffer RI, Kurosaki TT, Harrah CH, Chance JM, Filos S (1982). Measurement of functional activities in older adults in the community. J Gerontol.

[30] Sanchez MADS, Correa PCR, Lourenço RA (2011). Cross-cultural Adaptation of the "Functional Activities Questionnaire - FAQ" for use in Brazil. Dement Neuropsychol.

[31] Assis LO, Paula JJ, Assis MG, Moraes EN, Malloy-Diniz LF (2014). Psychometric properties of the Brazilian version of Pfeffer's Functional Activities Questionnaire. Front Aging Neurosci.

[32] Bustamante SEZ, Bottino CMC, Lopes MA, Azevedo D, Hototian SR, Litvoc J (2003). Combined instruments on the evaluation of dementia in the elderly preliminary results. Arq Neuropsiquiatr.

[33] Sanchez MAS, Lourenço RA (2009). Informant Questionnaire on Cognitive Decline in the Elderly (IQCODE): cross-cultural adaptation for use in Brazil. Cad Saude Publica.

[34] Perroco TR, Bustamante SEZ, Moreno MPQ, Hototian SR, Lopes MA, Azevedo D (2009). Performance of Brazilian long and short IQCODE on the screening of dementia in elderly people with low education. Int Psychogeriatr.

[35] Lino VTS, Pereira SRM, Camacho LAB, Ribeiro ST, Buksman S (2008). Cross-cultural adaptation of the Independence in Activities of Daily Living Index (Katz Index). Cad Saude Publica.

[36] Chaves MLF, Camozzato AL, Godinho C, Kochhann R, Schuh A, Almeida VL (2007). Validity of the clinical dementia rating scale for the detection and staging of dementia in Brazilian patients. Alzheimer Dis Assoc Disord.

[37] Morris JC (1993). The Clinical Dementia Rating (CDR) current version and scoring rules. Neurology.

[38] Ministério da Saúde (BR) (2013). Portaria nº 1.298, de 21 de novembro de 2013. Aprova o Protocolo Clínico e Diretrizes Terapêuticas da Doença de Alzheimer. Diário Oficial da União.

[39] Caramelli P, Marinho V, Laks J, Coletta MVD, Stella F, Camargos EF (2022). Treatment of Dementia. Arq Neuropsiquiatr.

[40] Jessen F, Amariglio RE, Buckley RF, Flier WM, Han Y, Molinuevo JL (2020). The characterisation of subjective cognitive decline. Lancet Neurol.

[41] Rabin LA, Smart CM, Amariglio RE (2017). Subjective Cognitive Decline in Preclinical Alzheimer's Disease. Annu Rev Clin Psychol.

[42] Molinuevo JL, Rabin LA, Amariglio R, Buckley R, Dubois B, Ellis KA (2017). Implementation of subjective cognitive decline criteria in research studies. Alzheimers Dement.

[43] Harten AC, Mielke MM, Swenson-Dravis DM, Hagen CE, Edwards KK, Roberts RO (2018). Subjective cognitive decline and risk of MCI The Mayo Clinic Study of Aging. Neurology.

[44] César-Freitas KG, Suemoto CK, Power MC, Brucki SMD, Nitrini R (2022). Incidence of dementia in a Brazilian population: The Tremembé Epidemiologic Study. Alzheimers Dement.

[45] Reisberg B, Shulman MB, Torossian C, Leng L, Zhu W (2010). Outcome over seven years of healthy adults with and without subjective cognitive impairment. Alzheimers Dement.

[46] Rönnlund M, Sundström A, Adolfsson R, Nilsson L-G (2015). Subjective memory impairment in older adults predicts future dementia independent of baseline memory performance: Evidence from the Betula prospective cohort study. Alzheimers Dement.

[47] Mitchell AJ, Beaumont H, Ferguson D, Yadegarfar M, Stubbs B (2014). Risk of dementia and mild cognitive impairment in older people with subjective memory complaints: meta-analysis. Acta Psychiatr Scand.

[48] Snitz BE, Lopez OL, McDade E, Becker JT, Cohen AD, Price JC (2015). Amyloid-ß Imaging in Older Adults Presenting to a Memory Clinic with Subjective Cognitive Decline: A Pilot Study. J Alzheimers Dis.

[49] Perrotin A, Mormino EC, Madison CM, Hayenga AO, Jagust WJ (2012). Subjective cognition and amyloid deposition imaging: a Pittsburgh Compound B positron emission tomography study in normal elderly individuals. Arch Neurol.

[50] Amariglio RE, Mormino EC, Pietras AC, Marshall GA, Vannini P, Johnson KA (2015). Subjective cognitive concerns, amyloid-ß, and neurodegeneration in clinically normal elderly. Neurology.

[51] Buckley RF, Maruff P, Ames D, Bourgeat P, Martins RN, Masters CL (2016). Subjective memory decline predicts greater rates of clinical progression in preclinical Alzheimer's disease. Alzheimers Dement.

[52] Studart A, Nitrini R (2016). Subjective cognitive decline The first clinical manifestation of Alzheimer's disease?. Dement Neuropsychol.

[53] Petersen RC, Smith GE, Waring SC, Ivnik RJ, Tangalos EG, Kokmen E (1999). Mild cognitive impairment: clinical characterization and outcome. Arch Neurol.

[54] Petersen RC (2004). Mild cognitive impairment as a diagnostic entity. J Intern Med.

[55] Winblad B, Palmer K, Kivipelto M, Jelic V, Fratiglioni L, Wahlund LO (2004). Mild cognitive impairment--beyond controversies, towards a consensus: report of the International Working Group on Mild Cognitive Impairment. J Intern Med.

[56] Morris JC (2012). Revised criteria for mild cognitive impairment may compromise the diagnosis of Alzheimer disease dementia. Arch Neurol.

[57] Portet F, Ousset PJ, Visser PJ, Frisoni GB, Nobili F, Scheltens P (2006). Mild cognitive impairment (MCI) in medical practice: a critical review of the concept and new diagnostic procedure. Report of the MCI Working Group of the European Consortium on Alzheimer's Disease. J Neurol Neurosurg Psychiatry.

[58] Albert MS, DeKosky ST, Dickson D, Dubois B, Feldman HH, Fox NC (2011). The diagnosis of mild cognitive impairment due to Alzheimer's disease: recommendations from the National Institute on Aging-Alzheimer's Association workgroups on diagnostic guidelines for Alzheimer's disease. Alzheimers Dement.

[59] American Psychiatric Association (2013). Diagnostic and statistical manual of mental disorders.

[60] World Health Organization (2019). International Classification of Diseases 11th Revision (ICD-11).

[61] Gold DA (2012). An examination of instrumental activities of daily living assessment in older adults and mild cognitive impairment. J Clin Exp Neuropsychol.

[62] Busse A, Hensel A, Gühne U, Angermeyer MC, Riedel-Heller SG (2006). Mild cognitive impairment: Long-term course of four clinical subtypes. Neurology.

[63] Farias ST, Mungas D, Reed BR, Harvey D, DeCarli C (2009). Progression of mild cognitive impairment to dementia in clinic- vs community-based cohorts. Arch Neurol.

[64] Manly JJ, Tang M, Schupf N, Stern Y, Vonsattel JPG, Mayeux R (2008). Frequency and course of mild cognitive impairment in a multiethnic community. Ann Neurol.

[65] Plassman BL, Langa KM, Fisher GG, Heeringa SG, Weir DR, Ofstedal MB (2008). Prevalence of cognitive impairment without dementia in the United States. Ann Intern Med.

[66] Di Carlo A, Lamassa M, Baldereschi M, Inzitari M, Scafato E, Farchi G (2007). CIND and MCI in the Italian elderly: frequency, vascular risk factors, progression to dementia. Neurology.

[67] Overton M, Pihlsgard M, Elmstahl S (2019). Prevalence and Incidence of Mild Cognitive Impairment across Subtypes, Age, and Sex. Dement Geriatr Cogn Disord.

[68] Petersen RC, Roberts RO, Knopman DS, Geda YE, Cha RH, Pankratz VS (2010). Prevalence of mild cognitive impairment is higher in men The Mayo Clinic Study of Aging. Neurology.

[69] Ward A, Arrighi HM, Michels S, Cedarbaum JM (2012). Mild cognitive impairment disparity of incidence and prevalence estimates. Alzheimers Dement.

[70] Godinho C, Camozzato AL, Onyszko D, Chaves ML (2012). Estimation of the risk of conversion of mild cognitive impairment of Alzheimer type to Alzheimer's disease in a south Brazilian population-based elderly cohort: the PALA study. Int Psychogeriatr.

[71] Chaves ML, Camozzato AL, Godinho C, Piazenski I, Kaye J (2009). Incidence of mild cognitive impairment and Alzheimer disease in Southern Brazil. J Geriatr Psychiatry Neurol.

[72] César KG, Brucki SMD, Takada LT, Nascimento LFC, Gomes CMS, Almeida MCS (2016). Prevalence of Cognitive Impairment Without Dementia and Dementia in Tremembé, Brazil. Alzheimer Dis Assoc Disord.

[73] Frota NAF, Nitrini R, Damasceno BP, Forlenza O, Dias-Tosta E, Silva AB (2011). Critérios para o diagnóstico de doença de Alzheimer. Dement Neuropsychol.

[74] World Health Organization (2019). International Classification of Diseases 10th Revision (ICD-10).

[75] Boff MS, Sekyia FS, Bottino CMC (2015). Prevalence of dementia among brazilian population: systematic review. Rev Med.

[76] Prince M, Bryce R, Albanese E, Wimo A, Ribeiro W, Ferri CP (2013). The global prevalence of dementia: a systematic review and metaanalysis. Alzheimers Dement.

[77] Oliveira D, Jun Otuyama L, Mabunda D, Mandlate F, Gonçalves-Pereira M, Xavier M (2019). Reducing the Number of People with Dementia Through Primary Prevention in Mozambique, Brazil, and Portugal: An Analysis of Population-Based Data. J Alzheimers Dis.

[78] Allegri RF (2020). Moving from neurodegenerative dementias, to cognitive proteinopathies, replacing "where" by "what".... Dement Neuropsychol.

[79] Grinberg LT, Nitrini R, Suemoto CK, Ferretti-Rebustini REL, Leite REP, Farfel JM (2013). Prevalence of dementia subtypes in a developing country: a clinicopathological study. Clinics.

[80] Suemoto CK, Ferretti-Rebustini REL, Rodriguez RD, Leite REP, Soterio L, Brucki SMD (2017). Neuropathological diagnoses and clinical correlates in older adults in Brazil: A cross-sectional study. PLoS Med.

[81] Elahi FM, Miller BL (2017). A clinicopathological approach to the diagnosis of dementia. Nat Rev Neurol.

[82] Geschwind MD (2016). Rapidly Progressive Dementia. Continuum.

[83] Dias NS, Barbosa IG, Kuang W, Teixeira AL (2020). Depressive disorders in the elderly and dementia: An update. Dement Neuropsychol.

[84] Bennett S, Thomas AJ (2014). Depression and dementia: cause, consequence or coincidence?. Maturitas.

[85] Livingston G, Huntley J, Sommerlad A, Ames D, Ballard C, Banerjee S (2020). Dementia prevention, intervention, and care: 2020 report of the Lancet Commission. Lancet.

[86] Antinori A, Arendt G, Becker JT, Brew BJ, Byrd DA, Cherner M (2007). Updated research nosology for HIV-associated neurocognitive disorders. Neurology.

[87] Rodrigues RA, Oliveira RL, Grinsztejn B, Silva MTT (2013). Validity of the International HIV dementia scale in Brazil. Arq Neuropsiquiatr.

[88] Gascón MRP, Vidal JE, Mazzaro YM, Smid J, Marcusso RMN, Capitão CG (2018). Neuropsychological Assessment of 412 HIV-Infected Individuals in São Paulo, Brazil. AIDS Patient Care STDS.

[89] 2019 American Geriatrics Society Beers Criteria(r) Update Expert Panel (2019). American Geriatrics Society 2019 Updated AGS Beers Criteria(r) for Potentially Inappropriate Medication Use in Older Adults. J Am Geriatr Soc.

[90] Caramelli P, Teixeira AL, Buchpiguel CA, Lee HW, Livramento JA, Fernandez LL (2011). Diagnosis of Alzheimer's disease in Brazil: Supplementary exams. Dement Neuropsychol.

[91] Petersen RC, Aisen P, Boeve BF, Geda YE, Ivnik RJ, Knopman DS (2013). Mild cognitive impairment due to Alzheimer disease in the community. Ann Neurol.

[92] Thijssen EH, La Joie R, Wolf A, Strom A, Wang P, Iaccarino L (2020). Diagnostic value of plasma phosphorylated tau181 in Alzheimer's disease and frontotemporal lobar degeneration. Nat Med.

[93] Loeffler T, Schilcher I, Flunkert S, Hutter-Paier B (2020). Neurofilament-Light Chain as Biomarker of Neurodegenerative and Rare Diseases With High Translational Value. Front Neurosci.

[94] Palmqvist S, Janelidze S, Quiroz YT, Zetterberg H, Lopera F, Stomrud E (2020). Discriminative Accuracy of Plasma Phospho-tau217 for Alzheimer Disease vs Other Neurodegenerative Disorders. JAMA.

[95] Lesman-Segev OH, La Joie R, Iaccarino L, Lobach I, Rosen HJ, Seo SW (2021). Diagnostic Accuracy of Amyloid versus 18F-Fluorodeoxyglucose Positron Emission Tomography in Autopsy-Confirmed Dementia. Ann Neurol.

